# A Lab-on-a-Tube Biosensor Combining Recombinase-Aided Amplification and CRISPR-Cas12a with Rotated Magnetic Extraction for *Salmonella* Detection

**DOI:** 10.3390/mi14040830

**Published:** 2023-04-09

**Authors:** Shangyi Wu, Jing Yuan, Ai Xu, Lei Wang, Yanbin Li, Jianhan Lin, Xiqing Yue, Xinge Xi

**Affiliations:** 1 College of Food Science, Shenyang Agricultural University, Shenyang 110866, China; wushangyi@stu.syau.edu.cn; 2 Key Laboratory of Agricultural Information Acquisition Technology, Ministry of Agriculture and Rural Affairs, China Agricultural University, Beijing 100083, Chinaxuai@cau.edu.cn (A.X.); wanglei123@cau.edu.cn (L.W.); jianhan@cau.edu.cn (J.L.); 3 Department of Biological and Agricultural Engineering, University of Arkansas, Fayetteville, AR 72701, USA; yanbinli@uark.edu

**Keywords:** lab-on-a-tube biosensor, large-volume sample, RAA/ CRISPR-Cas12a, rotated magnetic extraction, *Salmonella* detection

## Abstract

Background: Foodborne pathogenic bacteria threaten worldwide public health, and simple bacterial detection methods are in urgent need. Here, we established a lab-on-a-tube biosensor for simple, rapid, sensitive, and specific detection of foodborne bacteria. Methods: A rotatable Halbach cylinder magnet and an iron wire netting with magnetic silica beads (MSBs) were used for simple and effective extraction and purification of DNA from the target bacteria, and recombinase-aided amplification (RAA) was combined with clustered regularly interspaced short palindromic repeats/CRISPR-associated proteins12a(CRISPR-Cas12a) to amplify DNA and generate fluorescent signal. First, 15 mL of the bacterial sample was centrifuged, and the bacterial pellet was lysed by protease to release target DNA. Then, DNA-MSB complexes were formed as the tube was intermittently rotated and distributed uniformly onto the iron wire netting inside the Halbach cylinder magnet. Finally, the purified DNA was amplified using RAA and quantitatively detected by the CRISPR-Cas12a assay. Results: This biosensor could quantitatively detect *Salmonella* in spiked milk samples in 75 min, with a lower detection limit of 6 CFU/mL. The fluorescent signal of 10^2^ CFU/mL *Salmonella* Typhimurium was over 2000 RFU, while 10^4^ CFU/mL *Listeria* monocytogenes, *Bacillus* cereus, and *E. coli* O157:H7 were selected as non-target bacteria and had signals less than 500 RFU (same as the negative control). Conclusions: This lab-on-a-tube biosensor integrates cell lysis, DNA extraction, and RAA amplification in one 15 mL tube to simplify the operation and avoid contamination, making it suitable for low-concentration *Salmonella* detection.

## 1. Introduction

Foodborne pathogens are one of the key factors resulting in foodborne diseases, which threaten global healthcare systems and cause both economic and social problems [1,2]. *Salmonella* Typhimurium is among the most frequently reported foodborne pathogens worldwide, which often appears in meat, eggs, milk, and ready-to-eat products [3]. *Salmonella* Typhimurium matching human outbreak cases were identified in buttermilk tanks at a Ferrero Corporate plant in December 2021 and January 2022. Polymerase chain reaction (PCR), enzyme-linked immunosorbent assay (ELISA), biosensors, and other rapid detection methods have been employed for regular screening, which have shown a shorter detection time than the plate culture method [4,5,6]. Metal nanomaterial-based microfluidic chips and surface-enhanced Raman scattering-based sensors were able to detect as low as 60 CFU/mL *Salmonella* in 45 min or 70 CFU/mL in 2 h [7,8]. However, these rapid methods still rely on specific equipment and professional operators, and false positives are not easily avoided. In order to prevent and control *Salmonella* Typhimurium outbreaks and reduce the burden of foodborne diseases, establishment of sensitive, accurate, and convenient methods is essential.

For pathogen identification, molecular diagnostics is the critical method. Recently, nucleic acid isothermal amplification methods, such as recombinase polymerase amplification (RPA) [9,10,11] and loop-mediated isothermal amplification (LAMP) [12], are simple, rapid, and low-cost methods, which have been considered as attractive alternatives to conventional PCR. RAA is another common isothermal amplification method, which includes three major enzymes consisting of single-strand DNA-binding protein (SSB), recombinase, and DNA polymerase, and is proposed to detect *Salmonella* [13]. The recognition mechanism is as follows: first, target sequences in the sample are combined with the paired recombinase and specific primers (designed according to target sequences); then, SSB binds to the resulting displaced single-stranded DNA and prevents the immediate formation of double-stranded DNA; and the amplification is achieved by DNA polymerase, elongating the DNA in a short time. However, it is essential to control undesired nonspecific amplification (e.g., false positives). Secondary amplification can avoid nonspecific amplification, but the superiority of isothermal amplification is affected by extra time and reagent [14]. Thus, the development of time-saving and accurate methods remains a challenge.

Clustered regularly interspaced short palindromic repeats/CRISPR-associated protein (CRISPR-Cas) systems are widely used for genome editing. In recent years, highly specific Cas proteins have been developed to control nonspecific signal of molecular diagnosis [15]. A CRISPR-Cas system includes Cpf1 endonuclease (Cas12a), CRISPR RNA (crRNA), and protospacer adjacent motif (PAM). A Cas12a-crRNA complex can recognize and bind targeted double-stranded DNAs (dsDNAs) or single-stranded DNAs (ssDNAs) with a PAM for RNA-guided DNA cleavage [16]. A Cas12a-crRNA-target DNA complex has both cis- and trans-cleavage activities, which can produce the breaks of targeted dsDNAs or ssDNAs and cleavage nonspecific ssDNAs [17,18]. Nowadays, CRISPR-Cas systems have been often combined with nucleic acid amplification for sensitive nucleic acid detection [19,20,21]. Yue developed a droplet Cas12a assay, which allowed as low as 17.5 copies/μL DNA to be reliably quantified [22]. Zhi designed a CRISPR-Cas12a-based molecular diagnostic method, and the detection limit for *C. jejuni* in the food matrix was 1.2 and 0.12 CFU/mL after 24 and 48 h incubation, respectively [10]. Du et al. developed a droplet digital polymerase chain reaction technology that was able to detect 5 CFU/mL *Salmonella* in 8 h, and it took 5 h in the pretreatment containing culture enrichment (4 h) and an immunomagnetic separation step (1 h) [23]. Chun et al. developed a novel molecular diagnostic platform based on LAMP to detect 100 CFU/mL *Salmonella* in about 3 h, and the pretreatment took about 2 h [24]. These studies successfully achieved DNA detection at low concentrations. However, they still needed independent pretreatment steps consisting of bacterial lysis, DNA extraction, purified DNA transfer, or even incubation. One of the major pretreatment steps was nucleic acid extraction, which often required trained professionals [25,26,27]. Hence, the integration of enrichment, lysis, extraction, purification, and amplification is urgently needed for point-of-care detection of foodborne bacteria. Additionally, compared with other Cas proteins, CRISPR-Cas 9 can be active and destruct dsDNAs, but maybe not be used with amplification methods because the amplified dsDNA products could be digested; CRISPR-Cas13a has a similar trans-cleavage mechanism as CRISPR-Cas12a, but the recognized target of the Cas13a assay is RNA, thereby the amplification product could only be detected through the transcription process [28]; Cas 14a targets single-stranded DNA, which could include extra steps since the RAA products from our amplification step are double-stranded DNA. Therefore, Cas12a was used in this study for bacterial DNA reorganization and ssDNA probe cleavage.

**Figure 1:**
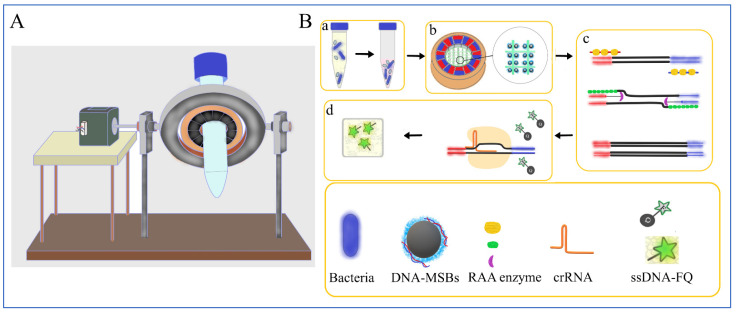
(**A**) The setup of the lab-on-a-tube biosensor; (**B**) the schematic of this lab-on-a-tube biosensor. (**a**) The yellow color and the pink color represent the milk sample and protease K with lysis buffer, respectively; (**b**) the relative position of MSBs and the iron wire netting; (**c**) the schematic diagram of RAA; (**d**) the schematic diagram of Cas12a-crRNA-target DNA complex active non-specific cleavage.

In this study, we developed an integrated biosensor for *Salmonella* detection in a 15 mL sample with high specificity. The setup of this biosensor is shown in Figure 1A. The biosensor is mainly composed of a 15 mL centrifugal tube, a Halbach cylinder magnet, an edge-fitted iron wire netting, and a stepper motor, and has functions including bacterial enrichment, cell lysis, DNA extraction, and RAA process. As shown in Figure 1B(a-d), a 15 mL sample is first centrifuged (10,000 rpm, 5 min) to collect the bacterial pellet. After the pellet is enzymatically lysed, the tube is placed into the center of the Halbach cylinder magnet and an iron wire netting is placed inside the tube; MSBs are added to adsorb the target DNA and form MSBs-DNA complexes, and the assembled device is rotated every 2 s to capture the MSBs-DNA complexes on the iron wire netting. Then, MSBs-DNA complexes are washed by the washing buffer containing ethanol, and the target DNA is released by the Tris-EDTA buffer solution (pH = 8), followed by amplification of purified DNA by RAA. Finally, the amplified DNA is detected by the CRISPR-Cas12a assay, where the cleavage activity of Cas12a is triggered by crRNA, and the quantitatively shows the concentration of bacteria.

The main novelty of this work is the development of an easy DNA purification device, which combines a uniform rotated magnetic field with an inner iron wire netting to capture MSBs-DNA complexes automatically and effectively. The high sensitivity of this biosensor is mostly due to the high-capture efficiency of target DNA from a large-volume sample, and the integration of the operation steps into one single tube; the high selectivity of this biosensor is due to the high specificity of the crRNA we designed. The biosensor achieved a detection limit as low as 6 CFU/mL for *Salmonella* in 75 min.

## 2. Materials and Methods

### 2.1. Materials and Reagents

*Salmonella* Typhimurium (ATCC 14028) was used as the target bacteria, while *Escherichia coli* O157:H7 (ATCC 43888), *Listeria monocytogenes* (ATCC 13932), and *Staphylococcus aureus* (ATCC 25293) were used as the non-target bacteria. For nucleic acid extraction and purification, the magnetic universal genomic DNA extraction kit and 1 μm magnetic silica beads were purchased from Tiangen Biotech (Beijing, China), and magnetic silica beads of 180 nm and 650 nm were purchased from Allrun Nano (Shanghai, China). For nucleic acid amplification, the RAA reaction kit was purchased from Amp Future (Weifang, China). For fluorescent signal generation, EvaGreen fluorescent dye was purchased from Maokang Biotech (Shanghai, China), and Lba Cas12a protein was purchased from New England BioLabs (Ipswich, USA). For crRNA synthesis and purification, the HiYeild T7 in vitro RNA synthesis kit and the 5 min RNA purification kit were purchased from HaiGene Biotechnology (Harbin, China), and the Magicscript RNase inhibitor was obtained from Magigen (Guanzhou, China). The nucleic acid sequences in this study (Table S1) were from Sangon Biotechnology (Shanghai, China).

### 2.2. The Device Design

Development of a simple DNA extraction and purification device was important for the following nucleic acid detection. In this study, a 15 mL spin centrifugal tube, a Halbach cylinder magnet, and an edge-fitted iron wire netting were fabricated and assembled to mix the sample and separate MSBs-DNA complexes from the sample for DNA amplification. As shown in Figure 1A, the Halbach cylinder magnet was fixed by an aluminum holder (orange ring) and driven by a stepper motor that intermittently span every 2 s with 20 rpm to form a rotated parallel magnetic field inside the magnet. In Figure 1B(c), the iron wire netting (iron wire diameter: 0.2 mm, square grid: 3 × 3 mm) coated with polyethylene (PE) were cut and fitted into the centrifugal tube. After the 15 mL centrifugal tube was coaxially assembled in the center of the Halbach cylinder magnet, the iron wire netting was placed in the center of the tube. The Halbach cylinder magnet was rotated by the stepper motor (Yixing company 42BYGH34, Zhejiang, China) controlled by an Arduino Uno (Yabo company R3, Shenzhen, China).

### 2.3. crRNA Preparation

The crRNA was designed based on the feature of the PAM site of Cas12a protein and selected using the Zhang Lab crRNA tool (http://chopchop.cbu.uib.no/, accessed on 1 June 2022.). The crRNA contained 25 nucleotides complementary to the sites on the *Salmonella* invA gene’s conserved fragment sequence of 453–563. The crRNA was synthesized according to Yue’s experimental protocol with modifications [22]. Firstly, to synthesize transcription DNA templates containing a T7 promoter, the mixture of each pair of DNA-TF and DNA-TR (2 μM final concentration) and 48 μL of diethylpyrocarbonate (DEPC)-treated water (DEPC water) were denatured at 95 °C for 5 min and cooled to room temperature. Secondly, the crRNA was in vitro transcribed using the DNA templates and a HiYeild T7 invitro RNA synthesis kit and incubated at 37 °C overnight. To remove the DNA templates, 1 μL of RNase-free DNase I was added and incubated at 37 °C for 15 min, and then the product was heated at 75 °C for 5 min to inactivate the polymerases. Thirdly, the product was purified using a 5 min RNA purification kit, and the purity values (OD260/280, OD260/230) of the crRNA were quantified by Nanodrop-2000 (Thermo Fischer Scientific, Waltham, MA, USA).

### 2.4. DNA Amplification and Detection

The purified DNA samples were amplified using real-time qPCR and RAA. The qPCR assay was carried out using the CFX96 Real-Time PCR system (Bio-Rad, Hercules, CA, USA) with the following amplification program: 98 °C for 2 min, and then 40 cycles at 98 °C for 5 s and at 55 °C for 5 s. Each qPCR reaction contained 10 μL of SsoFast™ EvaGreen supermix, 1 μL of each primer (10 μM), 3 μL of the target DNA, and up to 20 μL of deionized (DI) water [29]. The sequences of the primers were referred to in previous publication [13].

The RAA reaction was performed at 39 °C for 15 min, and each RAA reaction contained 29.4 μL of the RAA enzyme buffer A, 2 μL of forward and reverse primers (final concentration of 0.4 μM each), 11.6 μL of the purified DNA sample, 2.5 μL of magnesium acetate, and up to 50 μL of DI water. The primers were designed based on the primer selection from the TwistAmp^®^ assay design manual (https://www.twistdx.co.uk/support/rpa-assay-design/, accessed on 1 June 2022.) to amplify the gene fragment covering the crRNA target sites on the *Salmonella* invA gene’s conserved fragment. All the samples were tested in triplicate.

We adapted a fluorophore–quencher (FQ) assay with the Cas12a-crRNA-target DNA complex, cleaving the ssDNA-FQ to generate fluorescent signals, which correlated with the amplificated target DNA concentration. The CRISPR-Cas cleavage assay contained 100 nM Cas12a, 100 nM crRNA, 400 nM ssDNA-FQ reporter, and 2 μL of NEBuffer 2.1 with 2 μL of substrate dsDNA and up to 20 μL of DEPC water. The ssDNA-FQ reporter was synthesized with poly-A nucleotides labeled with a reporter (FAM) at the 5′ end and a quencher (BHQ) at the 3′ end. The reaction was carried out in the CFX96 for 30 min.

### 2.5. Bacterial Detection

For bacterial detection using this device, 15 mL of the sample was centrifuged (10,000 rpm, 5 min) in a tube, and the supernatant was removed to collect the pellet. A total of 20 μL of proteinase K was added into the tube and incubated for 15 min to lyse the bacterial pellet. A total of 15 μL of MSBs (30 mg/mL, 1μm) was added into the lysate and incubated for 10 min to capture DNA. The main driving forces for DNA adsorption onto the silica layer of MSBs included dehydration effect, electrostatic interaction, and intermolecular hydrogen bonding [30]. The MSBs-DNA complexes thus formed were continually captured onto the iron wire netting while the sample was mixed in the rotating tube. Then, the MSBs-DNA complexes were washed using 900 μL of wash buffer containing 40% ethanol for 1 time to remove protein and then using 900 μL of wash buffer containing 80% ethanol for 1 time to remove salt, and the complexes were released using 50 μL of Tris-EDTA buffer. The purified DNA was finally amplified and detected by the RAA/ CRISPR-Cas assay.

### 2.6. Real Sample Detection

Milk samples were used for food matrix detection. The spiked milk samples were prepared according to China’s food safety national standards GB 4789.4-2016. Briefly, 25 mL of milk purchased from a local market was first verified as having no target bacteria by the gold standard culture plating; then, it was diluted with 225 mL of sterile phosphate buffer, homogenized with a stomacher, and stood for 15 min to obtain the supernatant. Finally, 13.5 mL of the supernatant was mixed with 1.5 mL of bacteria cells at different concentrations (8.7 × 10^0^ to 10^4^ CFU/mL), which were detected using both the biosensor and culture plating. The spiked milk samples’ detection protocol using the biosensor was the same as described in Section 2.5.

## 3. Results and Discussion

### 3.1. Simulation of Magnetic Field

**Figure 2:**
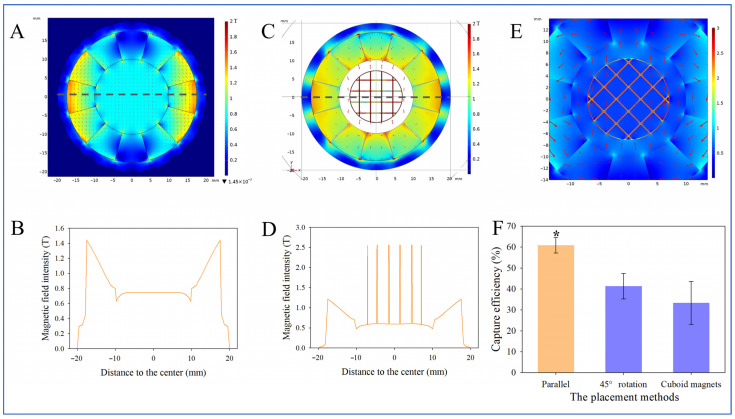
Magnetic field simulation and capture efficiency. (**A**) Simulation of magnetic field intensity within a Halbach cylinder magnet. (**B**) Magnetic field intensity along the horizontal line. Simulation of magnetic field intensity of the iron wire netting inside a Halbach cylinder magnet at parallel (**C**) and at 45° (**E**) using the placement methods. (**D**) Magnetic field intensity along the horizontal line. (**F**) Capture efficiency of the two placement methods and two cuboid magnets (N = 3). * indicates significant difference at *p* < 0.05.

A high-gradient magnetic field is essential for collecting MSBs and capturing nucleic acid. The Halbach cylinder magnet (inner diameter: 20.0 mm, outer diameter: 35.0 mm, and height: 10.0 mm) was comprised of twelve curved trapezoidal NdFeB magnets (grade: N52 and intersection angle: 60°) to produce a uniform magnetic field inside the magnet. Figure 2A,B show that a parallel magnetic field is formed inside the cylinder, and the intensity is 0.745 T. MSBs on this uniform magnetic field distributed uniformly and avoided aggregation, facilitating the capture of DNA.

According to the operation manual of the kit, it required 10 min of vortex to mix the DNA and MSBs; then, a commercial magnetic separator was used to separate the MSBs-DNA complexes. However, the magnetic separator could not handle a 15 mL sample as the magnetic field weakens rapidly with distance. The iron wire netting was placed in the tube and magnetized by the Halbach cylinder magnet to capture the MSBs efficiently. When the horizontal iron wires were parallel with the magnetic field lines, maximum intensity and gradient could be formed on the vertical iron wires. As shown in Figure 2C,D, the magnetic field intensity on the vertical iron lines increases over three times and generates a high magnetic gradient. MSBs and nucleic acid are captured around the vertical iron wires. The distances of the spikes correspond to the location of the iron netting. The iron netting was made of soft ferromagnetic material (cobalt–iron alloys) with a high magnetic induction and a high magnetic permeability. According to the equation B=μH, where *B* stands for magnetic field induction strength, *H* stands for magnetic field strength, and *μ* stands for magnetic permeability, the magnetic permeability of the iron netting is higher with the iron netting, and, thus, the magnetic field induction strength sharply increases where the iron netting is placed.

When none of the iron wires are parallel to the magnetic field lines, maximum intensity and gradient could be formed at the intersection points of the iron lines. As shown in Figure 2E, the magnetic field intensity at the intersection point of the iron wires reaches its highest point, where MSBs-DNA complexes are captured. In this case, MSBs-DNA complexes aggregate at the intersection points, and the amount of captured DNA decreases.

The two different placement methods mentioned above (i.e., the horizontal iron wires were placed parallel to the magnetic induction lines or rotated at 45°) and two cuboid magnets (length: 1.6 cm, width: 8 mm, height: 8 mm, and grade: N52), instead of the Halbach cylinder magnet, were tested for their capacities to capture the pure DNA sample (extracted from 15 mL of *Salmonella* sample at 8.7 × 10^6^ CFU/mL) for comparison. The DNA capture efficiency (*C_e_*) was calculated as the ratio of the DNA concentration from the biosensor (*C_b_*) to the added one (*C_a_*), i.e., *C_e_* = *C_b_*/*C_a_* × 100%. The results in Figure 2F show that the capture efficiency is 60.9%, 41.3%, and 33.3%, respectively (*p* < 0.05). The lower capture efficiency of the 45° rotation placement was because the maximum magnetic intensity was at the intersection points, and, therefore, MSBs accumulated at these intersection points, which hindered the adsorption of DNA molecules to the silicon surface. The cuboid magnets could not produce an intense and uniform magnetic field inside the tube (Figure S1), and MSBs might accumulate at the maximum-magnetic-intensity area on the iron wires or on the wall. Meanwhile, with the parallel placement method, MSBs uniformly distributed along the iron wires and, therefore, more silicon surface was exposed to capture DNA. Therefore, the iron wire netting was placed with the iron wires in parallel with the magnetic field lines. Statistical analysis was performed using SPSS (IBM SPSS 2 2, New York, NY, USA).

### 3.2. Selection of MSBs for DNA Extraction

**Figure 3:**
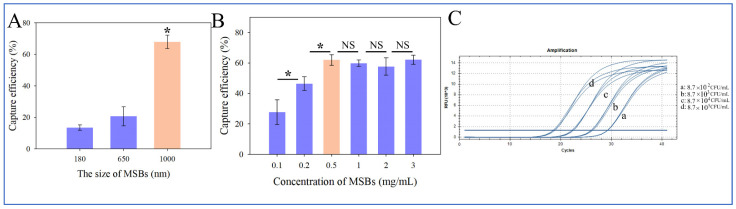
Optimization on amount and size of MSBs. (**A**) Capture efficiency for different sizes of MSBs (N = 3). (**B**) Capture efficiency for different concentrations of MSBs (N = 3). (**C**) Real-time qPCR results of DNA extracted from different concentrations of samples using the biosensor (N = 3). * indicates significant difference at *p* < 0.05. “NS” indicates no significant differences.

The amount and size of MSBs are important for efficient DNA extraction from the sample. The size of MSBs is vital for DNA extraction; therefore, 3 mg/mL of MSBs at different sizes of 180 nm, 650 nm, and 1 μm were applied to capture DNA, and the concentration was measured using Nanodrop 2000. As shown in Figure 3A, the capture efficiency of the 180 nm, 650 nm, and 1 μm MSBs is 13.5%, 20.7%, and 61.9%, respectively (*p* < 0.05). Since the magnetic force of MSBs increases with their size, 1 μm MSBs could be more stable on the magnetic iron wire, avoiding the loss of MSBs during the capture and washing processes. Meanwhile, the rotation and even dispersion can avoid the sediment of 1 μm MSBs.

Additionally, the amount of 1 μm MSBs (from 0.1 to 3 mg/mL) was investigated. As shown in Figure 3B, as the amount of MSBs increases from 0.1 to 0.5 mg/mL, the capture efficiency increases from 27.73% to 62.0% (*p* < 0.05). However, as the amount further increases to 3 mg/mL, there is no significant improvement in the capture efficiency; therefore, 0.5 mg/mL was selected as the tradeoff between high capture efficiency and reduced bead concentration. Hence, 0.5 mg/mL of MSBs was used for DNA extraction, which was six times less than the commercial recommended amount (15 μL in 200 ng/μL per 900 μL binding buffer, ~3.3 mg/mL). In general, a high amount of 1 μm MSBs could aggregate at the bottom of the tube; in this study, the rotation of the tube increased the chances for MSBs to mix with and capture DNA, and as a result, less amount of MSBs was required. Statistical analysis was performed using SPSS.

It is noted that MSBs cannot be reused [31] due to their reduced binding capacity and attachment to DNA particles even after DNA elution. The two-component DNA-binding silica matrix system with a regeneration buffer can save 70% of cost since the binding column can be reused at least 20 times [32], but this method still relies on centrifugation and could be hard to integrate on biosensors.

Real-time qPCR was used as the standard method to verify the purity of the target DNA using the lab-on-a-tube biosensor. In this method, 15 mL *Salmonella* samples at 8.7 × 10^2^–10^5^ CFU/mL were used in the test. The DNA extracted from these samples at the same concentration showed similar Ct values, and the DNA extracted from different concentrations showed significant difference in Ct values (Figure 3C), indicating that extracted DNA from this device could be used for the nucleic acid detection that followed. To evaluate the capture efficiency, the DNA extracted from the *Salmonella* samples (1.3 × 10^3^–10^6^ CFU/mL) using the nucleic acid extraction method recommended by the kit manual was set as the positive control. The Ct values of the four different bacterial concentrations obtained from the biosensor were consistent with the positive control according to the Bland–Altman analysis. As shown in Figure S2, most of the plots fall within the 95% limits of agreement, demonstrating that this biosensor collected a 15-fold larger volume and generated a similar signal as the 1 mL bacterial sample at a 15-fold higher concentration. This biosensor showed good performance in acquiring purified DNA. Thus, the pretreatment processing could significantly improve the sensitivity of the biosensor. Statistical analysis was performed using the MedCalc software (MedCalc 19.2.1, Ostend, Belgium).

### 3.3. Optimization of RAA-CRISPR/Cas12a Assay

First, to develop an “endpoint” RAA assay for quantitatively determining the amount of extracted DNA, 1 mL of *Salmonella* at different concentrations (1.3 × 10^2^–10^5^ CFU/mL) was used to optimize the incubation time. The reaction was set at 39 °C for 10, 15, 20, and 25 min in triplicate. As shown in Figure S3, the highest sensitivity is achieved with an incubation time of 15 min, and a good linear relationship (R^2^ = 0.99) is observed with the four concentrations tested. As the incubation time further increases, the linearity between bacterial concentration and fluorescent signal decreases; the amplification curve tends to reach a plateau at 25 min; there is no significant difference. Thus, the incubation time was set to 15 min in the RAA assay.

Secondly, the specificity of the RAA-CRISPR/Cas12a system depends on the generated crRNA. Here, the crRNA candidate, which is a crRNA-1 targeting invA gene amplified product, and crRNA-2 with no complementary sites to target sequence were designed and evaluated by comparing real-time fluorescent intensity. Figure S4 shows the crRNA sequences designed according to the PAM sequence positions in the amplification sequence, and the sequences of the amplification fragment with the PAM sites are listed. All the crRNAs were synthesized and purified, and the purify values (OD ratios of 260/280 and 260/230) are shown in Table S2. The OD ratios of 260/280 of the RNAs were 1.91 and 1.97, which fell within the normal range of 1.6–2.0, indicating little contamination from proteins in the isolated RNA. Meanwhile, the OD ratios of 260/230 were 2.21 and 2.29, which were both larger than 2, indicating little contamination from phenol, thiocyanates, and other organic compounds. The crRNAs were successfully synthesized with good purity.

**Figure 4:**
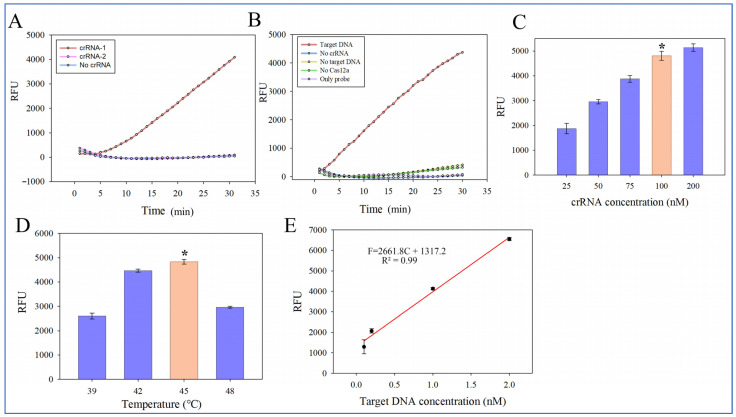
Optimization of the RAA-CRISPR/Cas12a assay. (**A**) Fluorescent intensity results for different crRNAs (N = 3). (**B**) Fluorescent intensity results of the RAA-CRISPR/Cas12a assay with missing elements (N = 3). (**C**) Fluorescent intensity results for different concentrations of crRNAs (N = 3). (**D**) Fluorescent intensity results for different CRISPR reaction temperatures (N = 3). (**E**) The calibration curve of the biosensor for synthetic fragments (N = 3). * indicates significant difference at *p* < 0.05.

According to the Zhang Lab crRNA tool, we selected crRNAs with high efficiency of 77. The two selected crRNAs, the target DNA (synthesized sequence, 1 nM), and DECP water as the negative control were used to verify the reaction efficiency. As shown in Figure 4A, the highest relative fluorescence unit (RFU) is obtained when crRNA-1 is used. For crRNA-2, as it does not contain complementary sites, the result of low fluorescence is similar to the negative control, indicating that it could not recognize the given DNA or cleave the ssDNA probe to generate signals. The high specificity indicates that non-specific cleavage could be activated only after specific crRNA identification of the target DNA is performed, and false positive results could be minimized. Therefore, crRNA-1 with the highest efficiency was selected in this study.

The RAA amplified product was used as the target DNA to verify the CRISPR-Cas12a assay. The real-time fluorescent CRISPR-Cas12a assay was performed at 45 °C for 30 min using the amplification product from the bacterial sample at 1.3 × 10^5^ CFU/mL. As shown in Figure 4B, the highest fluorescent signal is obtained when crRNA-1 is used, and the trans-cleavage and cis-cleavage activities of Cas12a can be triggered only when the target DNA and crRNA are both present, inducing the release of fluorescent ssDNA reporter probes to generate significantly enhanced fluorescent signals. Therefore, the system in this study could target the RAA amplification sequence and cleave the ssDNA reporter to generate signals.

Thirdly, the crRNA concentration (from 25 to 200 nM) and reaction temperature (from 39 °C to 48 °C) of the CRISPR-Cas12a assay were optimized to improve reaction efficiency. Synthetic fragments were used as the target DNA. As shown in Figure 4C, the fluorescent intensity at 100 nM crRNA is significantly different from that at 25, 50, and 75 nM crRNA, and fluorescent intensity increases from 1872 to 4804 as the concentration of crRNA increases from 25 to 100 nM. Although fluorescent intensity further increases as the concentration of crRNA increases from 100 nM to 200 nM, the increase is relative minor. The amount ratio of Cas and crRNA is 1:1, which is the same as reported in a previous study [33]. Therefore, 100 nM was used for crRNA in this study. Additionally, as shown in Figure 4D, fluorescent intensity increases from 2601 to 4831 when the CRISPR temperature increases from 39 °C to 45 °C, and it significantly decreases with a higher temperature (*p* < 0.05) since a higher temperature may inhibit enzyme activity. Consequently, the optimal CRISPR reaction temperature is 45 °C. This temperature is same as that from Yu’s study [22]. Statistical analysis was performed using SPSS.

Finally, to determine the detection range of the CRISPR-Cas12a assay, synthetic fragments at different concentrations (0.1 to 2 nM) corresponding to the selected crRNA were used under the optimal conditions. As shown in Figure 4E, a linear relationship between fluorescent intensity (*F*) and bacterial concentration (*C*) from 0.1 to 2 nM is observed and expressed by *F* = 2661.8lg*C* + 1317.2 (R² = 0.99).

### 3.4. Performance of the Biosensor for Salmonella Detection

**Figure 5:**
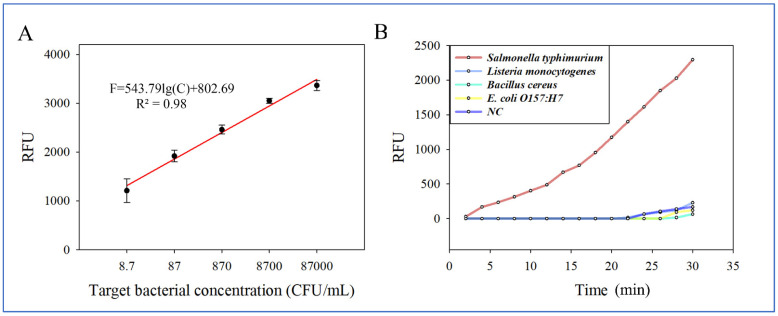
Performance of the biosensor. (**A**) The calibration curve of the biosensor (N = 3). (**B**) Specificity of the biosensor (N = 3).

To establish a calibration curve for the biosensor, 15 mL of the *Salmonella* sample at different concentrations from 8.7 × 10^0^ to 8.7 × 10^4^ CFU/mL were detected. The relationship between fluorescent intensity and bacterial concentration was evaluated under the optimal conditions by triplicate tests. As shown in Figure 5A, a linear relationship between fluorescent intensity (*F*) and bacterial concentration (*C*) from 8.7 × 10^0^ to 8.7 × 10^4^ CFU/mL of the 15 mL *Salmonella* samples is observed and expressed by *F* = 543.79lg*C* + 802.69 (R² = 0.98). The detection limit was determined as 2 CFU/mL using the signal-to-noise ratio. This biosensor has the advantages of larger sample volume, higher detection sensitivity, and/or shorter detection time than some recent studies (Table S3). The detection limit of this biosensor is lower than those of reported studies, which could be attributed to the following reasons: (1) DNA was extracted with high capture efficiency from a large volume because an iron wire netting placed inside a Halbach cylinder magnet generated a uniform magnetic field for DNA-MSBs complexes to be captured. The tube was rotated to mix and further avoid the aggregation of MSBs. (2) The signal was effectively amplified by the RAA/*CRISPR-Cas12a* assay since combining RAA with CRISPR-Cas12a increased sensitivity and specificity of this biosensor and avoided false positives.

The specificity of this biosensor was evaluated using *Salmonella* Typhimurium at 8.7 × 10^2^ CFU/mL as the target bacteria; *Listeria monocytogenes*, *Bacillus cereus,* and *E. coli* O157:H7 at 10^4^ CFU/mL as the non-target bacteria, and deionized water as the negative control (*NC*). As shown in Figure 5B, it is clear that the fluorescent intensity of the target bacteria is significantly higher than those of non-target bacteria, showing the good specificity of the biosensor. The specificity of the RAA-CRISPR/Cas12a system relies on the crRNA. Mismatches between the crRNA and its target sequence could impair the cleavage activity of the CRISPR/Cas12a complex, which makes CRISPR/Cas12a-based detection assays highly specific. All the pretreatments were conducted in the tube to avoid DNA contamination and, hence, avoided false positives.

To further evaluate the feasibility of this biosensor, 15 mL of pasteurized milk samples containing different concentrations of *Salmonella* Typhimurium (8.7× 10^0^–10^4^ CFU/mL) were tested. The recovery (*R*) was calculated as the ratio of the detected concentration (*C_d_*) to the added one (*C_a_*), i.e., *R*= *C_d_*/C_a_ × 100%. As shown in Table 1, the recovery rates for the target bacteria with different concentrations range from 79.2% to 114.3%, with an average of 96.0%, and the standard deviations range from 11.8% to 19.0%. The lower recovery rate at a lower concentration could be related to the high concentrations of impurities in milk, such as the high concentrations of β-casine, lactose, and fats, which can be absorbed on the surface of MSBs through electrostatic adsorption and other intermolecular force [34,35,36], impacting the DNA purification. With the concentration increase, the interruptions relatively decrease and can be ignored. Additionally, the amount of MSBs may influence DNA recovery too, and the optimal bacterial concentration in the milk sample could be between 870 and 8700 CFU/mL. This biosensor has a lower detection limit at about 6 CFU/mL in the 15 mL milk samples, and, therefore, we conclude that the biosensor can be used for *Salmonella* detection in large volume of pasteurized milk samples. Since this biosensor is easy to be assembled, it can be used for in-field detection. This biosensor can be used to detect *Salmonella* spp. since it targets the invA gene of the *Salmonella* genus.

The time for the whole detection procedure was about 75 min, including 5 min for bacterial pellet collection, 10 min for enzymic lysis, 10 min for DNA extraction, 5 min for washing, and 45 min for amplification and detection. Moreover, compared to some reported biosensors (0.1–1 mL), a large volume of 15 mL was processed in this biosensor, and this greatly improved the sensitivity without significantly increasing the processing time. The excellent performance of this biosensor could be attributed to the following aspects: (1) effective enrichment of the target bacteria from the 15 mL sample; (2) effective DNA extraction; (3) sensitive and highly specific DNA detection by the RAA/CRISPR-Cas12a method; (4) the protocols for bacterial enrichment, cell lysis, DNA capture, and target amplification were integrated on the same tube to eliminate contamination, with simple operation.

In this study, the RAA-CRISPR/Cas12a assay requires separate steps of target amplification and detection, in case the template DNA could be digested by Cas12a. Chen et al. [37] integrated a RAA-CRISPR/Cas12a system in a centrifugal microfluidic point-of-care test, but this device did not include the pretreatment step. Ma et al. [21] explored the colorimetric and photothermal features of AuNPs’ aggregation-to-dispersion change with CRISPR-Cas12a, and 1 CFU/mL *Salmonella* was successfully detected. However, this method did not integrate a nucleic acid extraction step. Jiang et al. [38] designed gold nanoparticle (AuNP) probes based on a magnetic pull-down colorimetric method to regulate the capture of AuNP probes and to visually analyze the CRISPR trans-cleavage reaction, with a detection limit of 50 RNA copies per reaction, while our biosensor still relies on a fluorescence detector. In future study, we will develop a biosensor using naked eye for quantification and integrate the separation and detection steps on the biosensor to handle more complex samples for point-of-care foodborne bacterial detection.

## 4. Conclusions

Herein, we developed a simple, rapid, sensitive, and specific lab-on-a-tube biosensor for *Salmonella* detection in a large-volume sample. An intermittently rotated centrifugal tube with a Halbach cylinder magnet and an iron wire netting was applied in this biosensor for efficient DNA extraction, and the DNA capture efficiency was above 60%. The selected crRNA effectively increased the sensitivity and specificity of this biosensor and avoided false positive. The lower detection limit of *Salmonella* was 2 CFU/mL in the 15 mL pure samples and 6 CFU/mL in the 15 mL spiked milk samples, and the detection process could be finished in 75 min. Moreover, bacterial enrichment, cell lysis, DNA extraction, and RAA amplification were all conducted in the same tube to simplify the operation and avoid contamination. Our future study will explore a portable lab-on-a-tube biosensor system including the signal detection system, making it suitable for point-of-care detection for *Salmonella*.

## Figures and Tables

**Table 1:** Detection of *Salmonella* Typhimurium in spiked milk samples (N = 3).

Added Concentration (CFU/mL)	Detected Concentration (CFU/mL)	Recovery (%)
8.7	7.1 ± 1.7	81.4 ± 19.0
87	94.9 ± 13.7	109.1 ± 15.7
870	995.0 ± 110.5	114.3 ± 12.7
8700	8329.0 ± 1025.6	95.7 ± 11.8
87,000	68,888.5 ± 13,373.5	79.2 ± 15.4

## Supplementary Materials

The following supporting information can be downloaded at https://www.mdpi.com/article/10.3390/mi14040830/s1. Table S1: Nucleic acid sequences used in this work; Table S2: The purify value of crRNA; Table S3: Comparison of this biosensor with some recently reported methods; Figure S1: Magnetic field simulation; Figure S2: The Ct values of this study compared with the method recommended by the manufacturer; Figure S3: Fluorescent intensity results of different incubation time; Figure S4: The PAM sequence positions.
