# Supplementary material for: A Lab-on-a-Tube Biosensor Combining Recombinase-Aided Amplification and CRISPR-Cas12a with Rotated Magnetic Extraction for Salmonella Detection
Source: Micromachines (Basel). 2023 Apr 9;14(4):830. doi: 10.3390/mi14040830 (PMC10144542; doi:10.3390/mi14040830)
Supplement: Supplementary file 1 [file micromachines-14-00830-s001.zip › micromachines-2309584-supplementary.pdf]

A Lab-on-a-Tube Biosensor Combining Recombinase Aided Amplification and CRISPR-Cas12a with Rotated Magnetic Extraction for *Salmonella* Detection

Table S1. Nucleic acid sequences used in this work.

Nucleic Acid ID	Sequences (5'-3')
RAA primer-F	GGTCCAGTTTATCGTTATTACCAAAGGTTCA
RAA primer -R	TTCAAATCGGCATCAATACTCATCTGTTTACCG
Complete nucleic acid sequence of RAA (111 bp)	GGTCCAGTTTATCGTTATTACCAAAGGTTCAGAACGTGTCGCGGAAGT CGCGGCCCGATTTTCTCTGGATGGTATGCCCGGTAAACAGATGAGTATT GATGCCGATTTGAA
PCR primer-F	ATTGGCGATAGCCTGGCGGTGGGTTTTGTTGT
PCR primer-R	TACCGGGCATACCATCCAGAGAAAATCGGGCCGC
DNA-TF-1	TAATACGACTCACTATAGGGTAATTTCTACTAAGTGTAGATCCGGGCAT ACCATCCAGAGAA
DNA-TR-1	TTCTCTGGATGGTATGCCCGGATCTACACTTAGTAGAAATTACCCTATA GTGAGTCGTATTA
crRNA-1	UAAUUUCUACUAAGUGUAGAUCCGGGCAUACCAUCCAGAGAA
DNA-TF-2	TAATACGACTCACTATAGGGTAATTTCTACTAAGTGTAGATCCGGGCAT ACCATCCAGAGAA
DNA-TR-2	TTCTCTGGATGGTATGCCCGGATCTACACTTAGTAGAAATTACCCTATA GTGAGTCGTATTA
crRNA-2	UAAUUUCUACUAAGUGUAGAUCCGGGCAUACCAUCCAGAGAA
ssDNA-FQ reporter	FAM-AAAAAAAAAAAAAAAAAAAA-BHQ

Table S2. The purify value of crRNA.

crRNA	OD 260/280	OD 260/230
crRNA-1	1.91±0.00	2.21±0.04
crRNA-2	1.97±0.01	2.29±0.02

Table S3. Comparison of this biosensor with some recently reported methods.

Methods	Targets	Total Time	Detection limit /Detection volume	Steps	References
LAMP	*Salmonella*	3 h	100 CFU/mL/ ~1.5mL	Lysis, extraction, amplification	[1]
Microfluidic& LAMP	SARS-CoV-2	1.5 h	20 copies/μL/ 50μL	RNA purification, amplification	[2]
Microfluidic & LAMP	*Enterococcus faecalis*	1 h	10 CFU/mL/ 0.5mL	Lysis, extraction, amplification	[3]
Digital PCR	Lung cancer cell L858R	1.2 h	10 copies/μL/ 20μL	Amplification	[4]
Digital PCR	*Salmonella*	8 h	0.2 CFU/mL/ 25mL	Enrichment, lysis, amplification	[5]
PCR	SARS-CoV-2	1.4 h	100 copies/mL/ 0.15mL	RNA purification, amplification	[6]
Electrochemical Aptamer	HBV	1.2 h	10000 copy/mL/ 1 mL	DNA extraction	[7]
RAA	*Staphylococcus aureus*	3.5 h	100 CFU/mL/ 0.5mL	DNA extraction, amplification	[8]
Surface-enhanced Raman scattering	*Salmonella*	2 h	70 CFU/mL/ 0.2mL	-	[9]
Colorimetric	*Salmonella*	0.8 h	60 CFU/mL/ 0.03mL	-	[10]
Electrochemistry	*Salmonella*	2 h	33 CFU/mL/ 0.5mL	-	[11]
This study	*Salmonella*	1.3 h	2 CFU/mL/ 15mL	Enrichment, lysis, extraction, purification, amplification	

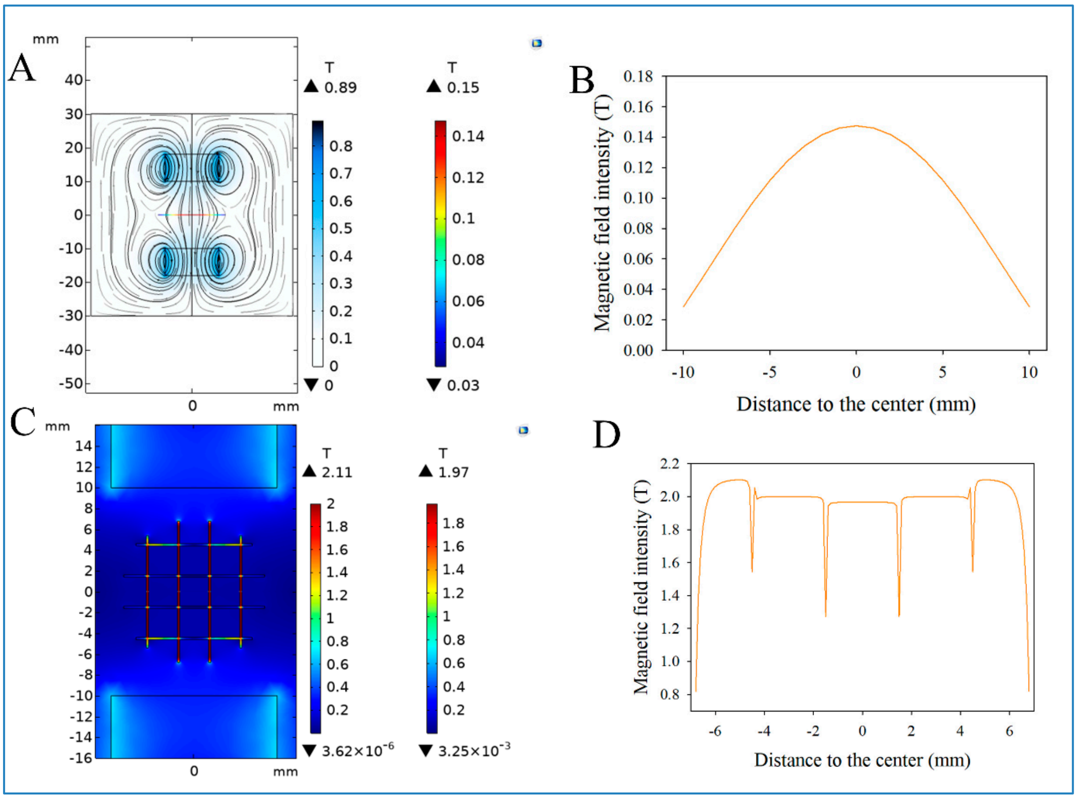

Figure S1. Magnetic field simulation (**A**) Simulation on the magnetic field intensity within two magnets. (**B**)Magnetic field intensity along the horizontal line. (**C**) Simulation on the magnetic field intensity of the iron wire netting inside two magnets at left second vertical line. (**D**)Magnetic field intensity of the iron wire netting inside two magnets at left second vertical line.

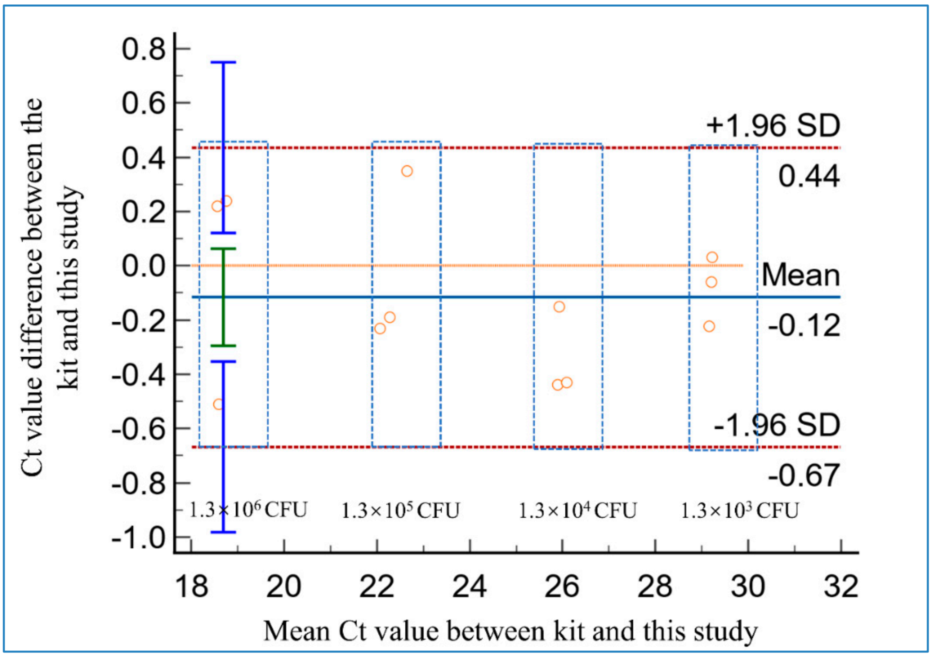

Figure S2. The Ct values of this study compared with the method recommended by the manufacturer (N = 3). The x-axis was the mean Ct value of the kit and this study, and the y-axis was the Ct value difference between the kit and this study (that is, the Ct value using the kit subtracted from the Ct value using the extraction method in this study) (N=3). The blue solid line indicated the mean of Ct value differences at different bacterial concentrations, and the red dashed lines indicated the mean differences ± 1.96 standard deviation (SD).

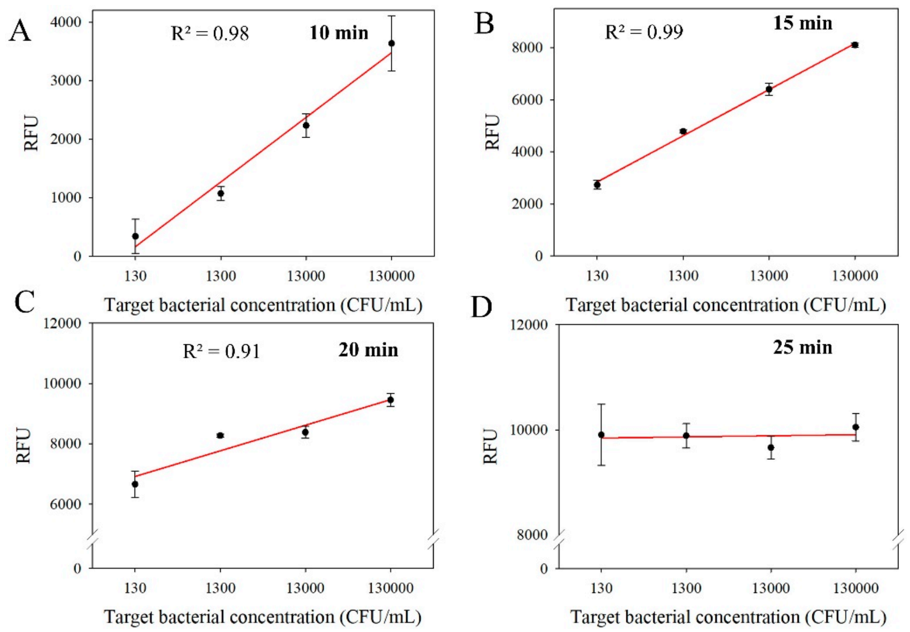

Figure S3. Fluorescent intensity results of different incubation time (N = 3).

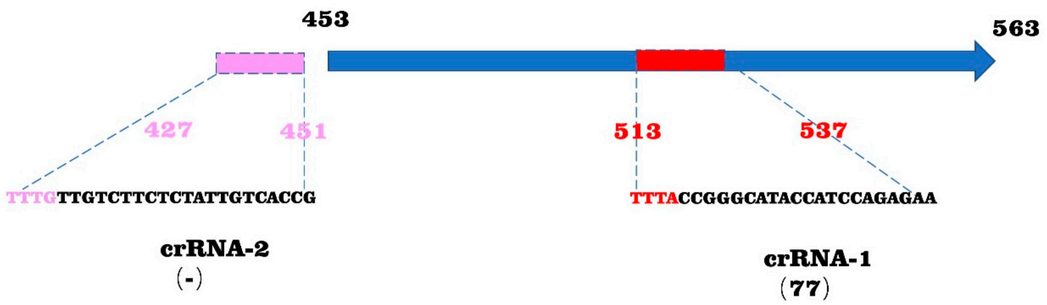

Figure S4. The PAM sequence positions (the TTTN form marked in pink and red, N represented A and G) in the amplification product (blue line).

References

1. Chun, H.J.; Kim, S.; Han, Y.D.; Kim, K.R.; Kim, J.-H.; Yoon, H.; Yoon, H.C. Salmonella Typhimurium Sensing Strategy Based on the Loop-Mediated Isothermal Amplification Using Retroreflective Janus Particle as a Nonspectroscopic Signaling Probe. *ACS Sensors* **2018**, *3*, 2261–2268.
2. Nguyen, H.V. Total Integrated Centrifugal Genetic Analyzer for Point-of-Care Covid-19 Testing with Automatic and High-Throughput Capability. *Sens. Actuators B Chem.* **2022**, *353*, 131088.
3. Gowda, H.N. Development of a Proof-of-Concept Microfluidic Portable Pathogen Analysis System for Water Quality Monitoring. *Science of the Total Environment* **2022**, *813*, 152556.
4. Cui, X. Fast and Robust Sample Self-Digitization for Digital PCR. *Anal. Chim. Acta* **2020**, *1107*, 127–134.
5. Du, M.; Li, J.; Liu, Q.; Wang, Y.; Chen, E.; Kang, F.; Tu, C. Rapid Detection of Trace Salmonella in Milk Using an Effective Pretreatment Combined with Droplet Digital Polymerase Chain Reaction. *Microbiological Research* **2021**, *251*, 126838, doi:10.1016/j.micres.2021.126838.
6. Liang, C. A Film-Lever Actuated Switch Technology for Multifunctional, on-Demand, and Robust Manipulation of Liquids. *Nat. Commun.* **2022**, *13*, 4902.
7. Li, J. Amplification-Free Smartphone-Based Attomolar HBV Detection. *Biosens. Bioelectron.* **2021**, *194*, 113622.
8. Xie, G. Recombinase Aided Amplification with Photoreactive DNA-Binding Dye for Rapid Detection of Viable Staphylococcus Aureus. *LWT* **2021**, *135*, 110249.
9. Lin, H.-Y.; Huang, C.-H.; Hsieh, W.-H.; Liu, L.-H.; Lin, Y.-C.; Chu, C.-C.; Wang, S.-T.; Kuo, I.-T.; Chau, L.-K.; Yang, C.-Y. On-Line SERS Detection of Single Bacterium Using Novel SERS Nanoprobes and A Microfluidic Dielectrophoresis Device. *Small* **2014**, *10*, 4700–4710, doi:10.1002/smll.201401526.
10. Man, Y.; Ban, M.; Li, A.; Jin, X.; Du, Y.; Pan, L. A Microfluidic Colorimetric Biosensor for In-Field Detection of Salmonella in Fresh-Cut Vegetables Using Thiolated Polystyrene Microspheres, Hose-Based Microvalve and Smartphone Imaging APP. *Food Chemistry* **2021**, *354*, 129578, doi:10.1016/j.foodchem.2021.129578.
11. Hou, Y.; Cai, G.; Zheng, L.; Lin, J. A Microfluidic Signal-off Biosensor for Rapid and Sensitive Detection of Salmonella Using Magnetic Separation and Enzymatic Catalysis. *Food Control* **2019**, *103*, 186–193, doi:10.1016/j.foodcont.2019.04.008.
